# A Rare Case of Coronary Artery Embolism in a Patient with d-Transposition of the Great Arteries with Prior Mustard Repair

**DOI:** 10.7759/cureus.2183

**Published:** 2018-02-12

**Authors:** Prince Sethi, Udit Bhatnagar, Kelly Steffen, Edgard Bendaly, Adam Stys

**Affiliations:** 1 Cardiology/internal Medicine, University of South Dakota Sanford School of Medicine, Sanford Usd Medical Center; 2 Sanford Cardiovascular Institute, University of South Dakota Sanford School of Medicine, Sanford Usd Medical Center; 3 Pediatric Cardiology, University of South Dakota Sanford School of Medicine, Sanford Usd Medical Center

**Keywords:** transposition of the great arteries, mustard repair, atrial fibrillation

## Abstract

The dextro-transposition of great arteries (d-TGA) is a rare, congenital, cyanotic heart disease and there is a paucity of data regarding long-term cardiovascular outcomes. We present a rare case of non-ST-elevation myocardial infarction (NSTEMI) in a patient with surgically repaired d-TGA. A 43-year-old male who had previously undergone a Mustard atrial switch palliative procedure presented with chest pain and diaphoresis and was diagnosed with NSTEMI. A coronary angiogram revealed a small, underdeveloped, left anterior descending and a large, left circumflex coronary artery with an acute embolic lesion. The embolic lesion was secondary to atrial fibrillation and was successfully treated with aspiration thrombectomy. This case highlights the variations in coronary anatomy in surgically repaired d-TGA and the importance of recognizing the potential for long-term complications in these cases.

## Introduction

The dextro-transposition of great arteries (d-TGA) is a rare, congenital, cyanotic heart malformation in which there is ventriculoarterial discordance, the aorta arises from the right ventricle, and the pulmonary trunk arises from the left ventricle [1]. This discordance creates two parallel circuits, which results in deoxygenated blood in the systemic circulation. This is physiologically incompatible with life in the absence of fetal shunts and requires very early intervention after birth. Atrial switch and arterial switch surgeries have been used for d-TGA, with the arterial switch being the preferred surgery nowadays [2-3]. The Mustard atrial switch procedure was the most frequently performed corrective surgery for patients with d-TGA from the 1960s to 1980s. There is a paucity of data regarding the long-term cardiovascular outcomes due to the rarity of the condition and the poor prognosis due to multiple long-term risks, including ventriculoarterial discordance and eventual systemic right ventricular (s-RV) failure. We present a case of non-ST-elevation myocardial infarction (NSTEMI) in a patient with d-TGA who had a Mustard procedure in childhood.

## Case presentation

A 43-year-old male with d-TGA corrected with a Mustard atrial switch procedure presented to the emergency department with angina for one day. He was diagnosed with d-TGA at the time of birth and had a palliative balloon atrial septostomy. He subsequently underwent a Mustard atrial switch repair at the age of two years. In this procedure, a baffle directed blood from the vena cava to the left ventricle and pulmonic venous blood to the right ventricle.

**Figure 1:**
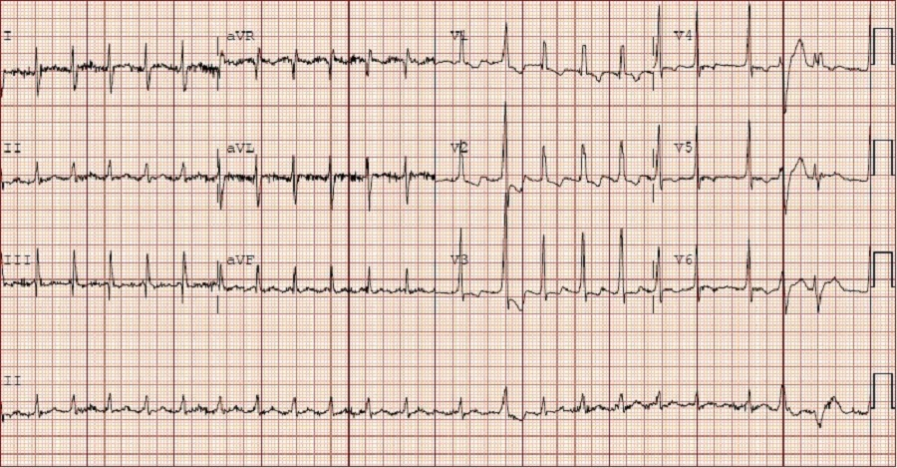
Electrocardiogram showing atrial fibrillation with non-specific ST-T wave changes.

**Figure 2:**
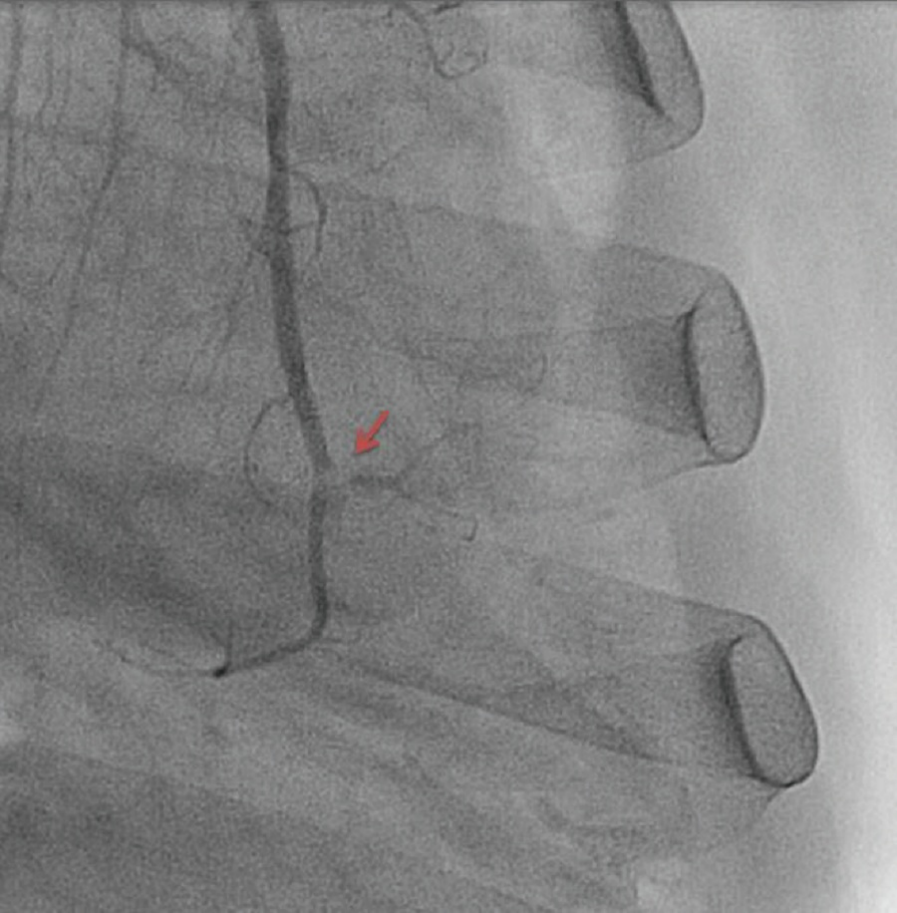
Left circumflex artery with acute embolic obstruction in the distal left circumflex artery, as shown by arrow

**Figure 3:**
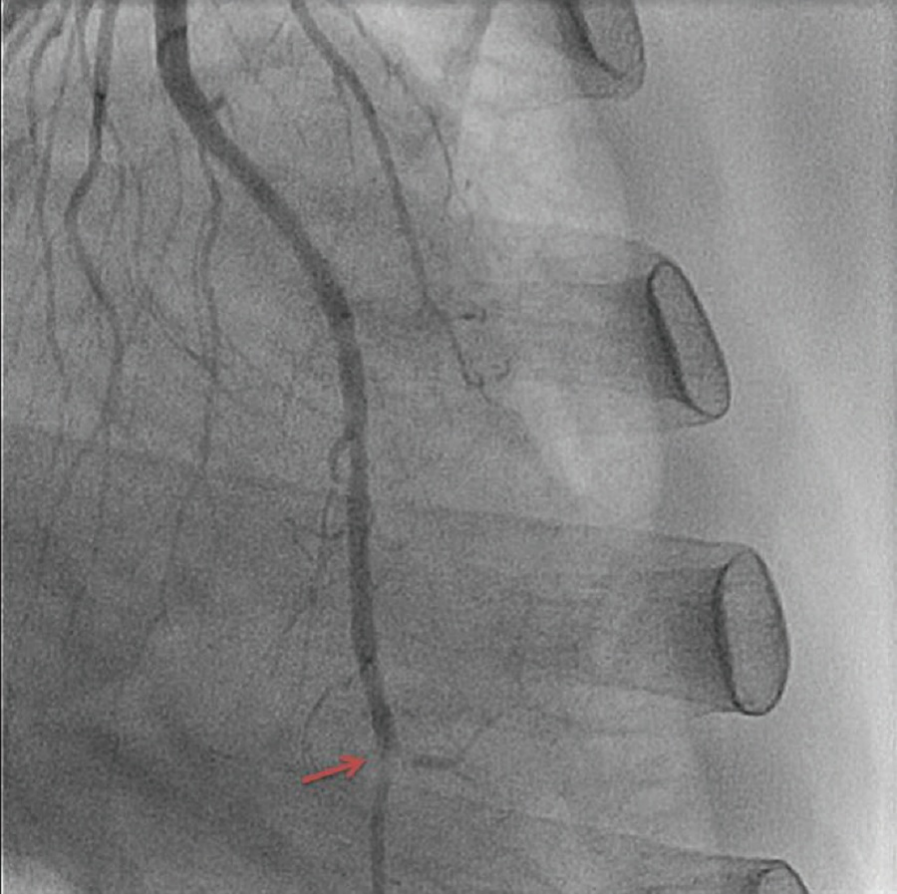
Left circumflex artery with acute embolic obstruction in the distal left circumflex artery, as shown by arrow

**Figure 4:**
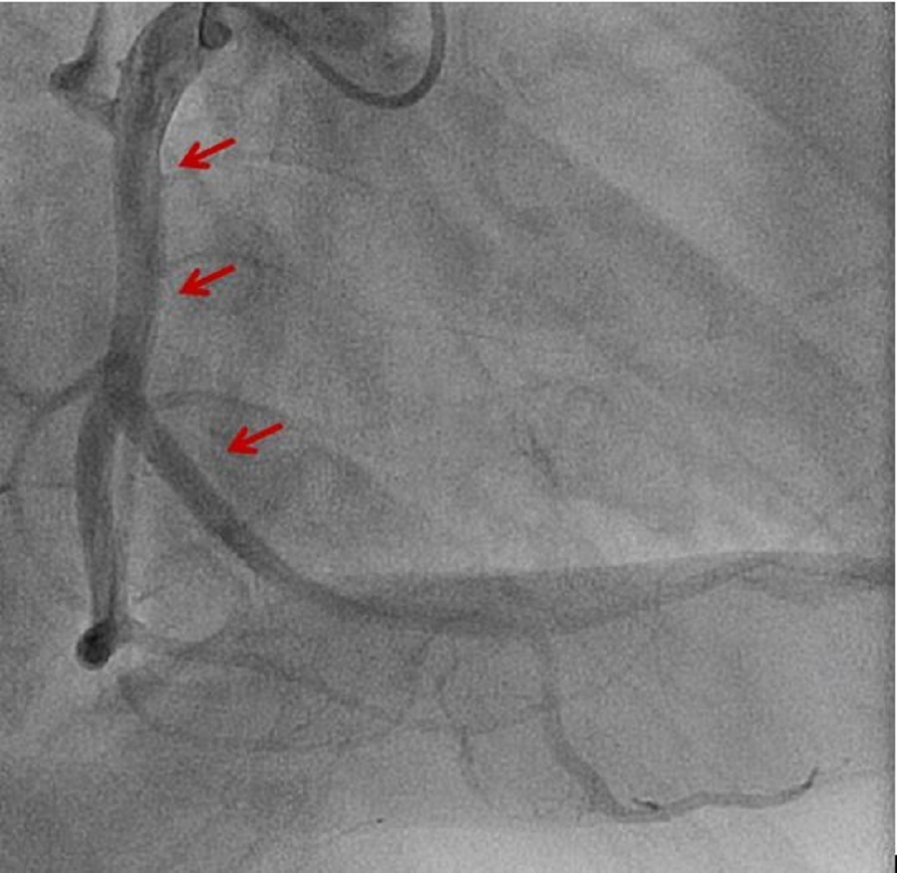
Large and patent right coronary artery

**Figure 5:**
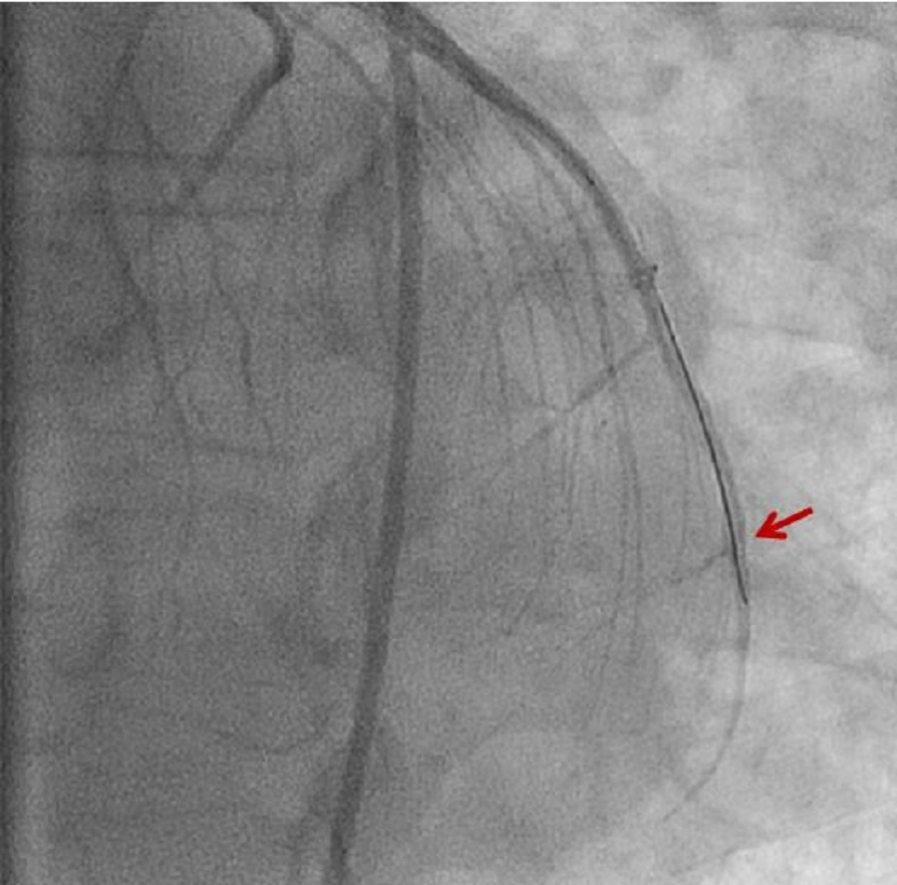
Post manual aspiration thrombectomy with resolution of embolic lesion and improved angiographic flow

The patient initially presented with a sudden onset of crescendo substernal chest pressure starting one day prior to presentation. He reported classic rest angina with substernal, pressure-like pain radiating to the back associated with dyspnea and diaphoresis. The electrocardiogram (ECG) showed atrial fibrillation with rapid ventricular rate and nonspecific ST-T changes (Figure 1). Troponin I was elevated at 3.82 ng/ml (normal: 0.00-0.03 ng/ml). The patient underwent an emergent coronary angiogram for non-ST-elevation myocardial infarction (NSTEMI). An evaluation of the left coronary system revealed a large left circumflex artery (LCx) with an embolus visualized in the distal segment (Figure 2, Figure 3). The left anterior descending artery (LAD) was small and underdeveloped. The right coronary artery (RCA), which supplied the majority of s-RV, was large and patent (Figure 4). Successful aspiration thrombectomy was performed for the LCx embolic lesion. Thereafter, good angiographic flow was documented (Figure 5). The patient’s symptoms resolved. Atrial fibrillation was treated with rate control using digoxin and metoprolol. Warfarin was used for anticoagulation. His hospital course was complicated by acute biventricular systolic failure requiring inotropic support, but he later stabilized on medical management. He was clinically improved at three months and is being followed in the congenital heart disease clinic.

## Discussion

TGA is a rare congenital cyanotic heart malformation with a prevalence of 4.73 per 10,000 live births [1]. In TGA, there is ventriculoarterial discordance, with the aorta arising from the right ventricle and the pulmonary trunk arising from the left ventricle [4]. This leads to the development of two parallel systems of pulmonary and systemic circulation instead of normal flow in series [4]. d-TGA is the more common variety of TGA compared to levo-TGA(l-TGA) [5]. In d-TGA, the aorta is positioned anterior to and on the right side of the pulmonary artery. d-TGA is a lesion, which is physiologically incompatible with life in the absence of a pulmonary-systemic circulation shunt in the presence of parallel pulmonary and systemic circuits. In utero, the fetus tolerates the defect well because of the presence of fetal shunts, including the ductus arteriosus and the foramen ovale.

Currently, the arterial switch procedure with the possibility to transfer the coronary arteries is preferred over the atrial switch, which allows the physiological left ventricle to function as the systemic left ventricle [2-3]. The Mustard atrial switch procedure was the most frequently performed corrective surgery for patients with d-TGA from the 1960s to the 1980s. The population who underwent the Mustard procedure is now aging.

TGA alone has known long-term complications, including tricuspid valve regurgitation, atrioventricular nodal conduction abnormalities, supraventricular arrhythmias, ventricular arrhythmias, and coronary artery abnormality [6-7]. The Mustard atrial switch procedure is also associated with the development of long-term complications, including atrial arrhythmia, baffle-related complications (such as baffle obstruction or leak), sinus node dysfunction, and right ventricular failure [2]. It is important that clinicians are aware of the long-term complications of the Mustard procedure in this aging population.

Coronary embolization is a rare cause of non-atherosclerotic acute coronary syndrome and this thrombo-embolic phenomenon is most commonly secondary to atrial fibrillation [8]. Myocardial infarction (MI) due to coronary embolism is seen more often in younger and female patients. Risk factors, such as diabetes, hypertension, smoking, and family history, are similar in non-atherosclerotic MI and atherosclerotic MI population groups except that hyperlipidemia is seen less frequently in patients with non-atherosclerotic MI [9]. The identification of coronary embolization as the cause of acute occlusive coronary disease is important because a modified interventional approach may be preferred such as aspiration thrombectomy and primary angioplasty with or without stent implantation [10]. The diagnosis of coronary embolism might influence subsequent management so as to address the underlying cause. Specifically, in this case, anticoagulation was used for atrial fibrillation.

In TGA, the usual origin of coronary arteries is seen in most patients. However, variation in coronary arteries may be present in one-fifth of the patients [7]. The use of computed tomography (CT) coronary angiography may provide valuable information in non-urgent situations regarding coronary anatomy especially in patients with post-surgically corrected congenital heart disease (CHD) allowing for the use of an appropriate interventional technique.

Our patient with d-TGA, who underwent the Mustard atrial switch procedure, presented with NSTEMI and atrial fibrillation with a rapid ventricular rate. He underwent a coronary angiogram. He met National Cerebral and Cardiovascular Center criteria for a definite coronary embolism with one major criterion (angiographic evidence of embolism without atherosclerotic disease) and two minor criteria (presence of atrial fibrillation and coronary angiography with <25% stenosis apart from the suspected embolic lesion) [8]. In our case, the patient also had a variation in coronary anatomy with a small and underdeveloped left anterior descending artery (LAD) supplying a small left ventricle (LV).

Variable presentations such as this one, where a patient with repaired TGA presented with NSTEMI caused by coronary embolism, should be kept in mind by physicians. This population is aging and may present with atypical findings due to the CHD repair procedure.

## Conclusions

Coronary artery embolism is a potential cause of acute coronary syndrome secondary to atrial fibrillation in a patient who has undergone an atrial switch procedure for d-TGA in childhood. In this subgroup of post-atrial switch patients, physicians should be aware of the variety of long-term complications due to the anatomical disparities, late complications of the disease process, and further accumulating age-related risk factors, such as atherosclerosis and hypertension. Such cases should be handled at experienced centers with interventional cardiologists and/or structural heart disease specialist.
